# Serum Midkine, estimated glomerular filtration rate and chronic kidney disease-related events in elderly women: Perth Longitudinal Study of Aging Women

**DOI:** 10.1038/s41598-020-71353-8

**Published:** 2020-09-02

**Authors:** Jeffrey Wang, Joshua R. Lewis, Elizabeth Byrnes, Germaine Wong, Warren D. Raymond, Kun Zhu, Graham R. Robertson, Wai H. Lim, Qi Cao, Richard L. Prince, Vincent W. Lee

**Affiliations:** 1 Centre for Transplant and Renal Research, University of Sydney at Westmead Institute of Medical Research, Westmead, 2145 Australia; 2 Centre for Kidney Research, Children’s Hospital at Westmead School of Public Health, Sydney Medical School, The University of Sydney, Sydney, 2006 Australia; 3 School of Medical and Health Sciences, Edith Cowan University, Joondalup, 6027 Australia; 4 Faculty of Health and Medical Sciences, The University of Western Australia, Perth, 6009 Australia; 5 Department of Endocrinology and Diabetes, Sir Charles Gairdner Hospital, Perth, 6009 Australia; 6 PathWest, QEII Medical Centre, Perth, 6009 Australia; 7 Lyramid Limited, Sydney, 2000 Australia; 8 Department of Renal Medicine, Sir Charles Gairdner Hospital, Perth, 6009 Australia

**Keywords:** Epidemiology, Chronic kidney disease, Prognostic markers

## Abstract

Midkine (MDK), a heparin-binding growth factor cytokine, is involved in the pathogenesis of kidney diseases by augmenting leukocyte trafficking and activation. Animal models and small case control studies have implicated MDK as a pathological biomarker in chronic kidney diseases (CKD), however this is yet to be confirmed in prospective human studies. In a prospective study of 499 elderly, predominantly Caucasian women aged over 70 years the association between serum MDK collected in 1998, and renal function change and the risk of CKD-related hospitalisations and deaths at 5 and 14.5 years, respectively, was examined. Baseline serum MDK was not associated with 5-year change in estimated glomerular filtration rate using the CKD Epidemiology Collaboration creatinine and cystatin C equation (Standardised β = − 0.09, 95% confidence interval − 3.76–0.48, p = 0.129), 5-year rapid decline in renal function (odds ratio = 0.97, 95% confidence interval 0.46–2.02, p = 0.927) or the risk of 14.5-year CKD-related hospitalisations and deaths (hazard ratio = 1.27, 95% confidence interval .66–2.46, p = 0.470) before or after adjusting for major risk factors. In conclusion, in this cohort of elderly women with normal or mildly impaired renal function, serum MDK was not associated with renal function change or future CKD-related hospitalisations and deaths, suggesting that MDK may not be an early biomarker for progression of CKD.

## Introduction

Chronic kidney disease (CKD) is a major global health concern, estimated to affect approximately 8–16% of the population worldwide^1^. End-stage kidney disease (ESKD), the most feared outcome for patients with CKD, is also associated with co-morbidities such as cardiovascular disease (CVD), and premature mortality^2^. The current standard measures such as estimated glomerular filtration rate (eGFR) and albuminuria are moderate performers at predicting disease progression to end-stage disease. Better estimates at earlier stage disease are needed to enable therapeutic interventions to minimise progression. While there have been a selection of biomarkers used to assess both innate kidney function and kidney damage^3^, most of these biomarkers have not been found sufficiently predictive of kidney function decline or disease progression to be used in routine clinical practice.

Midkine (MDK) is a heparin-binding cytokine most notably known for its role in embryonic development and cancer through promoting cell migration, survival, proliferation and angiogenesis^4–7^. In addition to its mitogenic properties, MDK also displays immunomodulatory functions which have been demonstrated in various inflammatory and autoimmune diseases^8–12^. Mechanism studies have shown that in the kidney, MDK promotes chemokine production and leukocyte trafficking post-kidney injury, and has been associated with increased renal inflammation, fibrosis and deterioration, indicating that MDK may play a role in mediating the pathogenesis of kidney diseases^13–17^.

A number of observational studies of individuals with acute kidney injury (AKI) or CKD have suggested MDK is clinically relevant with renal disease. One cross-sectional study in a CKD cohort showed that serum and urine MDK levels were inversely correlated with eGFR, and also elevated in patients with CKD stage 3 or above^18^. A few studies have reported that serum and urine MDK are predictive of AKI outcomes in post-cardiac surgery patients^19–21^. However, there is no prospective data available about the association between MDK and renal function decline and CKD development. This study aimed to determine the association between serum MDK and longitudinal changes in renal function; and CKD-related clinical events in a large cohort of elderly women with long-term clinical follow-up.

## Methods

All methods were carried out in accordance with relevant guidelines and regulations.

### Study population

The participants for this study were originally recruited in 1998 to a 5-year prospective, randomised, controlled trial of oral calcium supplements (1.2 g of elemental calcium daily), or an identical placebo to prevent osteoporotic fractures, the Calcium Intake Fracture Outcome Study (CAIFOS)^22^. Briefly, women aged over 70 years were recruited from the Western Australian general population by mail using the electoral roll, which is a requirement of Australian citizenship. Of the 5,586 who were approached, 1,500 were consented and recruited for the study. All participants were ambulant with an expected survival beyond 5 years and were not receiving any medication (including hormone replacement therapy) known to affect bone metabolism. Baseline disease burden, CVD risk and medications were comparable between these participants and the general population of similar age although these participants were more likely to be from higher socio-economic groups^22^. At the conclusion of CAIFOS, participants were subsequently included in additional follow-up studies for a further 10 years as the Perth Longitudinal Study of Aging in Women (PLSAW) https://www.lsaw.com.au, for a total follow-up period of 15 years. All participants that received placebo, or calcium treatment (calcium treatment code) from the CAIFOS were included for this study. Inclusion criteria for participants included all available exposure, confounding and outcome variables. Exclusion criteria included missing or invalid data on serum MDK, missing data on serum creatinine, cystatin C or both, and loss of follow-up data for clinical events (see Supplementary Fig. S1 and Supplementary Table S1 online).

### Baseline assessment

Baseline characteristics were assessed to determine potential confounding variables, which included age, body mass index (BMI), medical history and renal function. Treatment code (placebo or calcium) over the 5 years of the CAIFOS trial was also included as a covariate. Baseline medical history (diabetes and hypertension) was available from the demographic questionnaire, with the latter being verified by the participants’ general practitioners where possible. These data were coded using the International Classification of Primary Care-Plus (ICPC-Plus) method^23^. The coding methodology allows aggregation of different terms for similar pathological entities as defined by the ICD-10 coding system. Previous atherosclerotic vascular disease (ASVD) from 1980–1998 was determined from the Western Australian Hospital Morbidity Data Collection of primary discharge diagnosis^24^. The use of cardiovascular medications including blood pressure lowering agents and statins was assessed. Weight was assessed using digital scales with participants wearing light clothes and no shoes. Height was assessed using a stadiometer and the BMI was calculated using the following equation = weight (kg)/[height (m)]^2^. Estimated glomerular filtration rate (eGFR) was calculated using the Chronic Kidney Disease Epidemiology Collaboration (CKD-EPI) creatinine and cystatin C formula^25^.

### Biochemistry

Venous blood samples were collected between 0830 and 1030 h after overnight fasting at baseline and were stored at − 80 °C until assessment. In 2005, serum creatinine was measured from baseline serum samples using an isotope dilution mass spectrometry (IDMS) traceable Jaffe kinetic assay for creatinine (Hitachi 917 auto-analyzer, Roche Diagnostics GmbH, Mannheim Germany), whereas 5-year serum creatinine was measured in 2012 on the Architect ci16200 analyzer (Abbott Laboratories, Abbott Park, Ill., USA)^26^. Serum cystatin C was measured using a fully automated particle-enhanced immunoturbidimetric assay with Sentinel Diagnostics reagents (Sentinel CH, Milan, Italy) on the Architect ci 16200 System (Abbott Laboratories, Abbott Park, Ill., USA) according to the manufacturer’s instructions. The correlation coefficient (r^2^) between the machines was 0.998 with a Passing and Bablok slope of 0.966 and a Passing and Bablok intercept of 6.16 (n = 37)^26^.

### Midkine assay

MDK was measured in 2019 using a colourimetric enzyme-linked immunosorbent assay (human MDK SimpleStep ELISA kit—product ab193761: Abcam, Sydney, Australia)^27^ in baseline serum samples on the BioTek ELx800 Absorbance Microplate reader with BioTek Gen5 plate reading software (BioTek, Vermont, USA). The sensitivity (lowest detection limit) was 5 pg/mL, while the highest detection limit was 2096 pg/mL. The intra-assay and inter-assay coefficient of variance (CV) values were 5.57% and 14.26% respectively. For statistical purposes all samples below the lowest detection limit (n = 1) were assigned a value half of the lowest detection limit (2.5 pg/mL), while all values above the highest detection limit (n = 8) were assigned a value of the highest detection limit (2096 pg/mL). A change in the Abcam MDK SimpleStep ELISA kit lot led to high background absorbance for the standards (p < 0.001 for difference in kits) which could not be rectified after suggested amendments to the protocols from the manufacturer. We therefore excluded all results from the second batch of kit lots (n = 17 plates).

### Assessment of follow-up chronic kidney disease-related hospitalisations and deaths

The Western Australian Hospital Morbidity Data Collection of primary discharge diagnosis for hospitalisation and the Western Australian Deaths Registry have collected data on all hospital facilities and deaths occurring in Western Australia since 1980. At the commencement of this study in 1998 participants gave consent to access these registries, thus complete ascertainment for all CKD events causing hospitalisation and/or death for the 14.5 years from 1998 were available for all participants in this study. Deaths Registry data were coded from parts 1 and 2 of the death certificate and used the text fields from the death certificate to ascertain the cause(s) of deaths when necessary. These CKD events were linked to participants in this study via the Western Australian Data Linkage System (WADLS)^28^. Outcomes were identified using the International Classification of Diseases, Injuries and Causes of Death Clinical Modification (ICD-9-CM)^29^ and the International Statistical Classification of Diseases and Related Health Problems, 10th Revision, Australian Modification (ICD-10-AM)^30^. These codes included renal failure (ICD-9-CM codes 585.0–586.0, ICD-10-AM codes N18–19) from the primary discharge diagnoses.

### Statistical analysis

Baseline characteristics of the study participants were reported as mean (x̅) and standard deviation (SD) for continuous variables, frequencies (n) and percentages (%) for categorical variables, and median (x̃) and interquartile range (IQR) for variables not normally distributed. Baseline serum MDK was not normally distributed and was transformed using natural logarithm (ln). Serum MDK levels were used as either a continuous variable (ln-transformed, pg/mL), or a dichotomous category below or above the median value. Spearman’s correlation was used to assess univariate associations between serum MDK and continuous baseline clinical variables. Unadjusted and multivariable-adjusted linear regression analysis was used to assess the association between serum MDK with 5-year change in eGFR. The association between serum MDK and rapid decline in renal function, defined as an eGFR decline of greater than 15 mL/min/1.73 m^2^ at 5 years^31,32^, was assessed using unadjusted and multivariable-adjusted logistic regression analysis. The association between serum MDK with the occurrence of any CKD-related hospitalisation and death at 14.5 years was assessed using unadjusted and multivariable-adjusted Cox regression analysis. No violations of the proportional hazard assumptions were detected (p > 0.05). Two models of adjustment were used for all regression analyses: multivariable-adjusted and multivariable and baseline eGFR-adjusted. The multivariable-adjusted models included age, calcium treatment code, diabetes, and prevalent ASVD. Baseline eGFR was adjusted for separately as eGFR is a strong predictor of longitudinal kidney disease outcomes. Results were expressed as either odds or hazard ratio (OR or HR) with 95% confidence interval (CI) for the logistic and Cox regression models respectively. P values of less than 0.05 in two-tailed testing were considered statistically significant. All analyses were performed using IBM SPSS (version 24; SPSS Inc., Chicago, Ill., USA).

### Ethics and trial registration

The randomised, controlled trial was commenced and completed before the introduction of clinical trial registries, hence the trial was retrospectively registered in the Australian New Zealand Clinical Trials Registry ACTRN12615000750583. At baseline written informed consent was obtained from all participants for the study and follow-up of electronic health records. The Human Ethics Committee of the University of Western Australia approved the study protocol and consent form (Approval Number 05/06/004/H50). The Human Research Ethics Committee of the Western Australian Department of Health (DOHWA HREC) also approved the data linkage study (Approval Number #2009/24).

## Results

### Baseline characteristics

The median age of this study cohort was 75, with interquartile range (IQR) of 73 to 77 years. 45.9% and 18.0% of participants were maintained on anti-hypertensive and statin medications, respectively, while 6.8% and 13.8% of participants entered the study with prevalent diabetes and ASVD, respectively (Table 1). The mean (± SD) eGFR of the study cohort was 65.1 (± 12.7) mL/min/1.73 m^2^. 34.1% of participants had baseline eGFR less than 60 mL/min/1.73 m^2^.

**Table 1:** Baseline characteristics expressed based on the total study population or stratified by median of serum MDK (n = 499).

	All participants	MDK below median	MDK above median	p value
Number	499	250	249	
MDK, pg/mL	437 [328, 580]	329 [278, 387]	579 [491, 784]	
Age, years	75 [73, 77]	75 [73, 77]	75 [73, 78]	0.215
BMI, kg/m^2^	26.7 [24.0, 29.9]	27.1 [24.6, 30.3]	26.5 [23.3, 29.7]	0.097
Calcium treatment	257 (51.5)	136 (54.4)	121 (48.6)	0.194
Diabetes	34 (6.8)	13 (5.2)	21 (8.4)	0.152
Use of blood pressure lowering medication	229 (45.9)	110 (44.0)	119 (47.8)	0.395
Use of statin	90 (18.0)	43 (17.2)	47 (18.9)	0.626
Prevalent ASVD	69 (13.8)	31 (12.4)	38 (15.3)	0.355
Baseline CKD-EPI eGFR, mL/min/1.73m^2a ^	65.1 ± 12.7	65.4 ± 12.4	64.8 ± 13.0	0.605
Serum creatinine, mg/dL	0.90 ± 0.18	0.89 ± 0.17	0.90 ± 0.19	0.531
Serum cystatin C, mg/dL	1.07 ± 0.22	1.07 ± 0.21	1.08 ± 0.23	0.562

Results are expressed as mean ± SD, median and [IQR], or number and (%).

*SD* standard deviation, *IQR* interquartile range, *MDK* Midkine, *BMI* body mass index, *ASVD* atherosclerotic vascular disease, *CKD-EPI eGFR* Chronic Kidney Disease Epidemiology Collaboration estimated glomerular filtration rate.

^a^Measured in 422 participants using the CKD-EPI creatinine and cystatin C equation.

### Baseline serum MDK

Data on serum MDK at baseline was available in 499 women (see Supplementary Fig. S1 online). Serum MDK levels were not normally distributed (see Supplementary Fig. S2 online). The median [IQR] serum MDK level was 437 [328, 580] pg/mL. Baseline characteristics were stratified below or above the median serum MDK (Table 1). Neither age nor BMI were different between participants with serum MDK above or below the median, nor were correlated with serum MDK (see Supplementary Table S2 online).

A total of 422/499 (84.6%) of participants with data on serum MDK had data on baseline serum creatinine and cystatin C to allow calculation of eGFR (see Supplementary Fig. S1 online). Baseline eGFR did not differ between participants with serum MDK above or below the median (Table 1). Baseline eGFR, serum creatinine and cystatin C were not correlated with serum MDK (Supplementary Table S2 online).

Women with a history of ASVD had serum MDK levels significantly higher than those without (with ASVD median [IQR] 503 [385, 666 pg/mL], without ASVD 435 [323, 562 pg/mL], Mann–Whitney *U* test p = 0.016).

### Serum MDK and 5-year eGFR change/rapid decline in renal function

During follow-up there were 297/422 (70.4%) participants from this cohort with data on serum creatinine and cystatin C at 5 years for calculation of eGFR (see Supplementary Fig. S1 online). There were no associations between natural logarithm (ln)-serum MDK with 5-year eGFR change in unadjusted (Spearman’s rho = − 0.08, p = 0.297), multivariable-adjusted (Standardised β = − 0.09, 95% CI 3.25–0.40, p = 0.126) or multivariable and baseline eGFR-adjusted (Standardised β = − 0.09, 95% CI 3.28–0.28, p = 0.098) analyses. Similarly, below or above-median serum MDK was not associated with 5-year eGFR change in unadjusted (Spearman’s rho = − 0.06, p = 0.304), multivariable-adjusted (Standardised β = − 0.08, 95% CI 3.78–0.56, p = 0.146), or multivariable and baseline eGFR-adjusted (Standardised β = − 0.09, 95% CI 3.76–0.48, p = 0.129) analyses. Rapid decline in renal function was identified in 33/297 (11.1%) participants at 5 years. There were no associations between ln-serum MDK with 5-year rapid decline in renal function in unadjusted (OR 1.20, 95% CI 0.63–2.28, p = 0.573), multivariable-adjusted (OR 1.23, 95% CI 0.63–2.41, p = 0.538) or multivariable and baseline eGFR-adjusted (OR 1.26, 95% CI 0.64–2.46, p = 0.506) analyses. Similarly, below or above-median serum MDK was not associated with 5-year rapid decline in renal function in unadjusted (OR 0.96, 95% CI 0.46–1.98, p = 0.902), multivariable-adjusted (OR 0.96, 95% CI 0.46–2.01, p = 0.916) or multivariable and baseline eGFR-adjusted (OR 0.97, 95% CI 0.46–2.02, p = 0.927) analyses.

### Serum MDK and CKD-related hospitalisations and deaths

**Figure 1:**
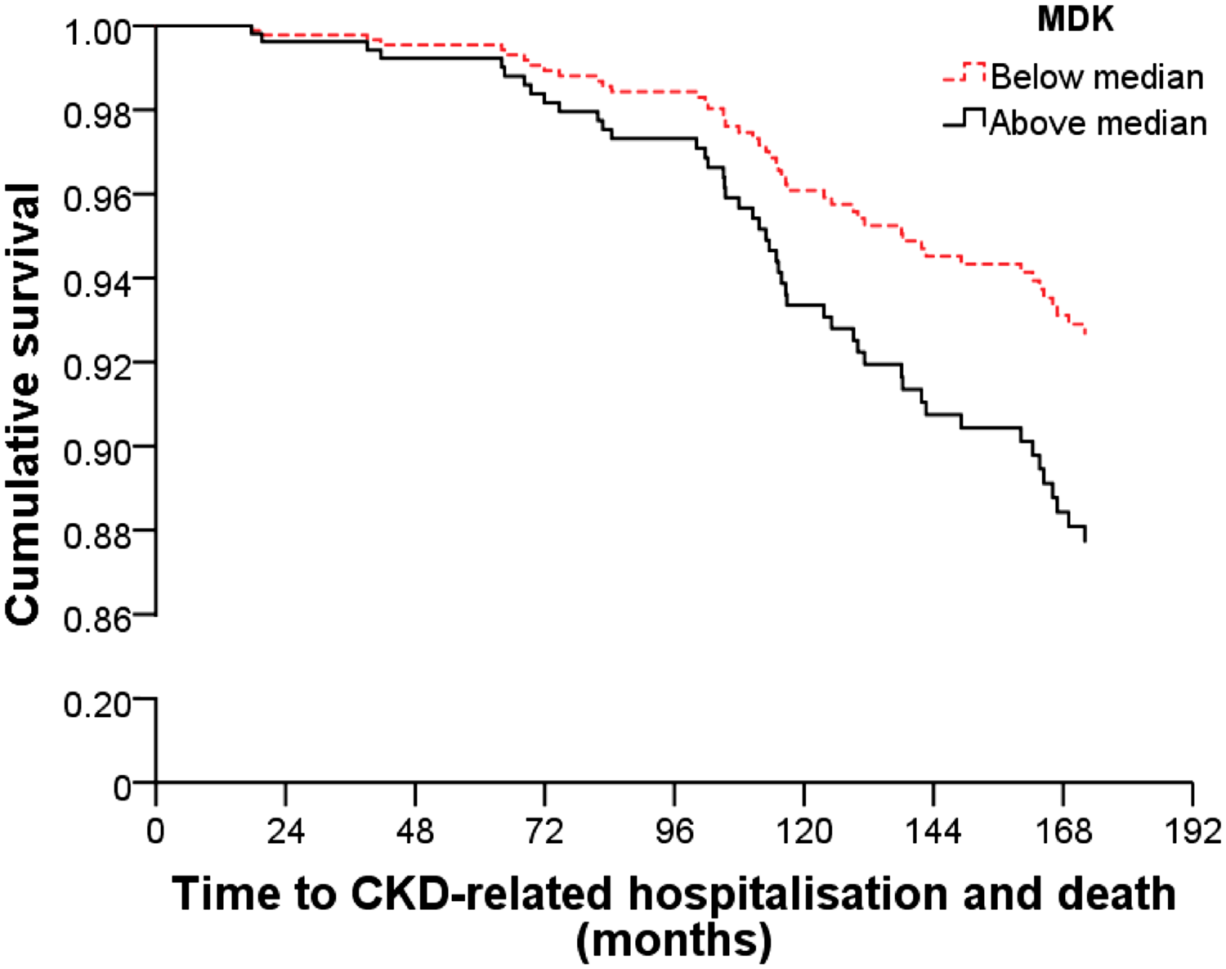
Multivariable-adjusted Cox proportional HR and 95% CI for 14.5-year CKD-related hospitalisations and deaths (n = 47) in elderly women stratified by serum MDK below (< 437 pg/mL, dashed red line, referent) or above (≥ 437 pg/mL, solid black line) the median. Multivariable model was adjusted for age, calcium treatment code, diabetes and prevalent atherosclerotic vascular disease. Multivariable HR 1.72, 95% CI 0.96–3.11, p = 0.071. *CKD* chronic kidney disease, *MDK* Midkine, *HR* hazard ratio, *CI* confidence interval.

CKD-related hospitalisations and deaths occurred in 47/499 (9.4%) participants over the 14.5 years of follow-up. The incidence of CKD-related hospitalisations and deaths in participants with below-median serum MDK was 18 (7.2%), and in those with above-median serum MDK it was 61% higher at 29 (11.6%) (Chi-squared test p = 0.089). In unadjusted Cox Proportional Hazard analysis the HR for participants with above-median serum MDK compared to those with below-median serum MDK was 1.82 (95% CI 1.01–3.28, p = 0.046). However in multivariable-adjusted, and multivariable and baseline eGFR-adjusted analyses the HR were 1.72 (95% CI 0.96–3.11, p = 0.071) and 1.27 (95% CI 0.66–2.46, p = 0.470), respectively (Table 2 and Fig. 1).

**Table 2:** Unadjusted and multivariable-adjusted Cox proportional hazard analysis of serum MDK in predicting 14.5-year CKD-related hospitalisations and deaths in elderly women.

	MDK below median (< 437 pg/mL)	MDK above median (≥ 437 pg/mL)	p value
Number of events (%)	18 (7.2)	29 (11.6)	
Unadjusted HR (95% CI)	**1.00 (referent)**	**1.82 (1.01, 3.28)**	**0.046**
Multivariable-adjusted HR (95% CI)	1.00 (referent)	1.72 (0.96, 3.11)	0.071
Multivariable and baseline eGFR-adjusted HR (95% CI)	1.00 (referent)	1.27 (0.66, 2.46)	0.470

Multivariable model was adjusted for age, calcium treatment code, diabetes and prevalent atherosclerotic vascular disease.

*MDK* Midkine, *CKD* chronic kidney disease, *eGFR* estimated glomerular filtration rate, *HR* hazard ratio, *CI* confidence interval.

Bold indicates statistical significance of the p value.

## Discussion

In this study, the baseline serum MDK levels were consistent to that previously reported in healthy populations^18,33^, and these levels of serum MDK were not associated with eGFR at baseline, 5-year eGFR decline, or 5-year rapid decline in renal function. Although we observed an association between above the median serum MDK levels with the risk of 14.5-year CKD-related hospitalisations and deaths in the unadjusted analysis, the association was no longer significant after adjusting for age, calcium treatment, diabetes and prevalent ASVD with or without baseline eGFR. These results suggest that circulating MDK levels consistent to that of previously reported healthy populations is not a useful biomarker in predicting renal function decline over time or CKD-related clinical events in the healthy, elderly female population.

The study results should be interpreted in context. Previous studies have reported associations between MDK and renal dysfunction and clinical outcomes in both AKI and CKD populations^19–21^. The discrepancy between our results to that of previous findings is likely due to the difference in study population characteristics – we examined an elderly yet healthy cohort with minimal comorbidities and disease burden comparable to the general population^34^, as opposed to other cohorts with established renal disease. These previous findings indicate that serum MDK may be a more sensitive biomarker associated with renal function changes when there is marked renal injury or dysfunction, and is therefore not as relevant to the early trajectory of renal function decline. We have also utilised serum cystatin C in addition to serum creatinine to measure eGFR in our study in contrast to previous studies which only utilised serum creatinine^18–21^, so the difference in findings may be due to fundamental differences in defining renal function.

Our findings may argue that MDK is not an early biomarker in chronic and age-related renal function changes and kidney disease outcomes, which is supported by both experimental and clinical evidence on the involvement of MDK in acute inflammation and kidney injury^14,15,20,21^. However, given that there are also a few studies addressing MDK in chronic inflammation and kidney disease cases^16–18^, this reflects the need to consider how MDK activity is regulated during the transition from acute to chronic renal inflammation and deterioration.

There were limitations with this study. There was no data available on proteinuria and albuminuria, so we were unable to determine if serum MDK was associated with these early indicators of kidney damage. The majority of serum MDK levels at baseline were within the normal range consistent with previous reports^18,33^. As such, we were unable to determine whether abnormal levels of serum MDK were associated with renal function decline or CKD-related clinical events. The proportions of the study population that experienced events for 5-year rapid decline in renal function and 14.5-year CKD-related hospitalisation and death were small (11.1% and 9.4%, respectively), which may have reduced our statistical power. There was loss of samples for data on serum MDK due to changing of the ELISA assay kit lots, which may also have reduced our statistical power. However, this is unlikely to have altered the magnitude of the associations as there were no significant differences between the characteristics (and therefore potential covariates in association) of the included and excluded cohorts (see Supplementary Table S3 online). Since we did not measure serum MDK levels longitudinally, we cannot exclude the possibility that MDK is a pathological biomarker in CKD. Finally, our study was limited to generally healthy, older predominantly Caucasian women so the findings may not apply to other demographic or disease populations, such as those with diabetes who are at increased risk of CKD.

The strengths of this study include the use of both serum creatinine and cystatin C for the measurement of eGFR, which improved our ability to more accurately define baseline kidney function and CKD outcomes. The study had a long follow-up period of 14.5 years, minimal loss to follow-up, and detailed prospective data collection using a comprehensive, population-based linkage system. This is also the first study to prospectively examine the relationship between MDK, renal function and clinical outcomes of CKD, and therefore provide insights to assess the clinical value of MDK as a viable prognostic biomarker.

In conclusion, baseline serum MDK is not associated with long-term renal function decline or CKD-related hospitalisations and deaths in healthy, elderly women with normal or mildly impaired renal function. Further studies are required to confirm the relationship between MDK and longitudinal renal function and CKD outcomes in populations with prevalent kidney disease.

## Supplementary information

Supplementary Information.

## Data Availability

The datasets generated during and/or analysed in this study can be viewed online at https://www.lsaw.com.au/ and are available from Prof Richard L. Prince at richard.prince@uwa.edu.au on reasonable request.
