# Supplementary material for: Serum Midkine, estimated glomerular filtration rate and chronic kidney disease-related events in elderly women: Perth Longitudinal Study of Aging Women
Source: Sci Rep. 2020 Sep 2;10:14499. doi: 10.1038/s41598-020-71353-8 (PMC7468100; doi:10.1038/s41598-020-71353-8)
Supplement: Supplementary file 1 — Supplementary Information. [file 41598_2020_71353_MOESM1_ESM.docx]

**Serum Midkine, estimated glomerular filtration rate and chronic kidney disease-related events in elderly women: Perth longitudinal study of aging women**

**Jeffrey Wang, Joshua R. Lewis, Elizabeth Byrnes, Germain Wong, Warren D. Raymond, Kun Zhu, Graham R. Robertson, Wai H. Lim, Qi Cao, Richard L. Prince, Vincent W. Lee**

1,500 participants recruited in 1998 for the CAIFOS study

(n = 1,500)

Excluded (n=317)

- No available serum for MDK assay
  n =317 (21.1%)

Assessed for valid data on serum MDK

n = 1,183 (78.9%)

Excluded (n = 684)

- No valid data on serum MDK
  n = 684 (45.6%)

Cross-sectional analysis

n = 499 (33.3%)

(n = 499)

Excluded (n = 77)

- Missing data on serum creatinine, cystatin C, or both n = 77 (5.1%)

Eligible for baseline (1998) eGFR analysis

n = 422 (28.1%)

(n = 499)

Excluded (n = 125)

- Missing data on serum creatinine, cystatin C, or both n = 125 (8.3%)

Eligible for 14.5-year follow-up incident CKD-related hospitalisation and death analysis

n = 499 (33.3%)

Eligible for 5-year follow-up eGFR analysis

n = 297 (19.8%)

(n = 499)

**Supplementary Figure S1:** Flowchart outlining the selection process of participants in this study. CAIFOS = Calcium Intake Fracture Outcome Study. MDK = Midkine. eGFR = estimated glomerular filtration rate. CKD = chronic kidney disease.


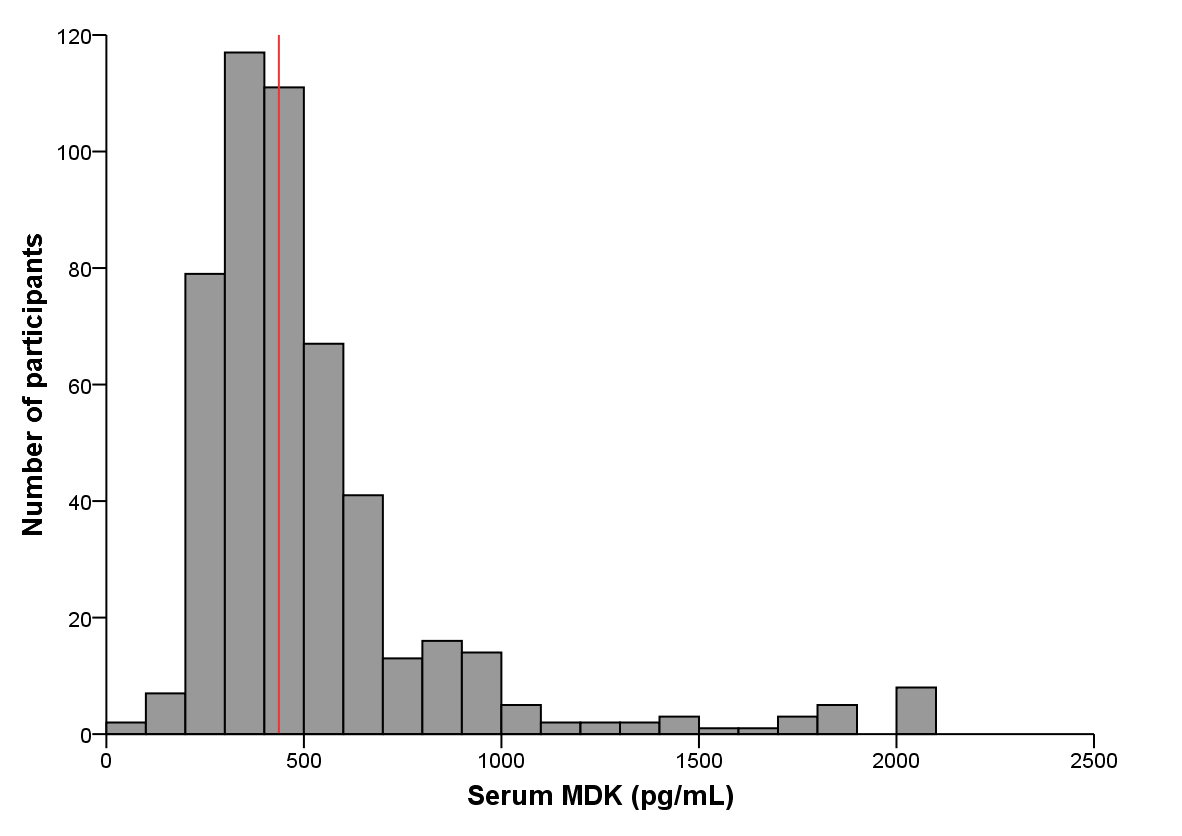


**Supplementary Figure S2.** Frequency distribution of serum MDK levels in the cohort. Median value indicated by the red reference line. Numbers on the *x axis* indicate serum MDK levels (pg/mL) in increments of 100 pg/mL. MDK = Midkine.

**Supplementary Table S1.** Patterns of missing data on measurements of eGFR in the cohort.

|  | **Baseline eGFR** | **5 years eGFR** | **N** | **%** |
| --- | --- | --- | --- | --- |
|  | X | X | 297 | 59.5 |
|  | X | . | 125 | 25.1 |
|  | . | X | 57 | 11.4 |
|  | . | . | 20 | 4.0 |
| Total | 422 | 354 | 499 | 100 |
| x = measured.  . = value missing.  eGFR = estimated glomerular filtration rate. | | | | |

**Supplementary Table S2.** Spearman’s rank correlation between serum MDK and clinical variables.

|  | **Number** | **Spearman’s rho (ρ)** | **p value** |
| --- | --- | --- | --- |
| Age, years | 499 | 0.060 | 0.184 |
| BMI, kg/m^2^ | 499 | -0.065 | 0.146 |
| Baseline CKD-EPI eGFR, mL/min/1.73m^2^ | 422 | -0.054 | 0.265 |
| Serum creatinine, mg/dL | 461 | 0.036 | 0.446 |
| Serum cystatin C, mg/dL | 429 | 0.055 | 0.255 |
| MDK = Midkine; BMI = body mass index; CKD-EPI eGFR = Chronic Kidney Disease Epidemiology Collaboration estimated glomerular filtration rate using creatinine and cystatin C. | | | |

**Supplementary Table S3.** Comparison of baseline characteristics between samples included and excluded for data on serum MDK in this study.

|  | **Samples with valid MDK values** | **Samples without valid MDK values** | **p value** |
| --- | --- | --- | --- |
| Number | 499 | 1001 |  |
| Age, years | 75.2 ± 2.7 | 75.2 ± 2.7 | 0.954 |
| BMI, kg/m^2^ | 27.2 ± 4.6 | 27.2 ± 4.8 | 0.880 |
| Calcium treatment | 257 (51.5) | 512 (51.2) | 0.912 |
| Diabetes | 34 (6.8) | 61 (6.1) | 0.593 |
| Use of blood pressure lowering medication | 229 (45.9) | 423 (42.3) | 0.186 |
| Use of statin | 90 (18.0) | 192 (19.2) | 0.587 |
| Prevalent ASVD | 69 (13.8) | 112 (11.2) | 0.141 |
| Baseline CKD-EPI eGFR, mL/min/1.73m^2^ | 65.1 ± 12.7 | 66.0 ± 13.2 | 0.226 |
| Data expressed as mean ± SD or number and (%). Continuous variables analysed by  One-way ANOVA, categorical variables analysed by χ^2^ test. SD = standard deviation; ANOVA = analysis of variance; MDK = Midkine; BMI = body mass index; ASVD = atherosclerotic vascular disease; CKD-EPI eGFR = Chronic Kidney Disease Epidemiology Collaboration estimated glomerular filtration rate. | | | |
